# Economic evaluation and costs of remote patient monitoring for cardiovascular disease in the United States: a systematic review

**DOI:** 10.1017/S0266462323000156

**Published:** 2023-04-28

**Authors:** Yunxi Zhang, Maria T. Peña, Lauren M. Fletcher, Lincy Lal, J. Michael Swint, Jennifer C. Reneker

**Affiliations:** 1 Department of Data Science, John D. Bower School of Population Health, University of Mississippi Medical Center, Jackson, MS 39216, USA; 2 Department of Management, Policy and Community Health, School of Public Health, The University of Texas Health Science Center at Houston, Houston, TX 78712, USA; 3 Brown University Library, Brown University, Providence, RI 02912, USA; 4 Rowland Medical Library, University of Mississippi Medical Center, Jackson, MS 39216, USA; 5 Center for Clinical Research and Evidence-Based Medicine, John P and Katherine G McGovern Medical School, The University of Texas Health Science Center at Houston, Houston, TX 77030, USA; 6 Department of Population Health Science, John D. Bower School of Population Health, University of Mississippi Medical Center, Jackson, MS 39216, USA

**Keywords:** Costs, Economic evaluation, Cost-effectiveness, Remote patient monitoring, Cardiovascular diseases, Telehealth, Telemedicine

## Abstract

**Background:**

Remote patient monitoring (RPM) has emerged as a viable and valuable care delivery method to improve chronic disease management. In light of the high prevalence and substantial economic burden of cardiovascular disease (CVD), this systematic review examines the cost and cost-effectiveness of using RPM to manage CVD in the United States.

**Methods:**

We systematically searched databases to identify potentially relevant research. Findings were synthesized for cost and cost-effectiveness by economic study type with consideration of study perspective, intervention, clinical outcome, and time horizon. The methodological quality was assessed using the Joanna Briggs Institute Checklist for Economic Evaluations.

**Results:**

Thirteen articles with fourteen studies published between 2011 and 2021 were included in the final review. Studies from the provider perspective with a narrow scope of cost components identified higher costs and similar effectiveness for the RPM group relative to the usual care group. However, studies from payer and healthcare sector perspectives indicate better clinical effectiveness of RPM relative to usual care, with two cost-utility analysis studies suggesting that RPM relative to usual care is a cost-effective tool for CVD management even at the conservative $50,000 per Quality-Adjusted Life-Year threshold. Additionally, all model-based studies revealed that RPM is cost-effective in the long run.

**Conclusions:**

Full economic evaluations identified RPM as a potentially cost-effective tool, particularly for long-term CVD management. In addition to the current literature, rigorous economic analysis with a broader perspective is needed in evaluating the value and economic sustainability of RPM.

## Introduction

Cardiovascular diseases (CVDs), as a group of disorders of the heart and blood vessels, are the leading cause of death globally for all sex, race, and ethnicity groups; approximately 17.9 million people die each year from CVDs (1). In the United States (US), about half of adults suffer from CVDs, including hypertension (2;3). Remote patient monitoring (RPM), as a patient-centered healthcare delivery method, has emerged for managing CVD at home (4;5). Integrated with technology for data transition, RPM patients collect their health information, for example, weight, blood pressure (BP), blood glucose, and heart rate, at home through personal health devices and send it to healthcare facilities so that medical providers at the point of care can closely monitor the patient health status (5;6). Many systematic reviews and meta-analyses have demonstrated short- and long-term effectiveness of RPM in improving chronic CVD care management, including better quality of life, faster clinical event detection, less re-hospitalization, and lower mortality rates (7–11). Recognizing the effectiveness of RPM in managing CVD, the American Heart Association (AHA) published guidance on RPM implementation to encourage the use of RPM for better CVD outcomes (4). Also, in 2018, the Center for Medicare & Medicaid Services (CMS) issued CPT codes to reimburse providers for delivering RPM services to patients, and further coverage for RPM services has been added in its recently proposed 2022 Physician Fee Schedule (12;13).

Aside from the effectiveness of managing CVD, it is critical to evaluate the related economic costs. According to an AHA report from 2018, the medical costs related to CVD are expected to more than triple by 2035 (14), which implies that the US is experiencing an unsustainable health expenditure growth for CVD. However, though the importance of economic sustainability of RPM for CVD management has gained attention, less has been systematically reviewed and synthesized across the studies conducted. Given the lack of clear information about the cost implications of RPM for CVD management and the complexity and uniqueness of the US healthcare system, this review aims to examine the economic cost and cost-effectiveness of RPM compared to usual care for CVD management in the US.

## Methods

This systematic review was conducted following the JBI methodology for systematic reviews of economic evidence in accordance with a published protocol (15;16), associated registration on PROSPERO (CRD42021270621), and written following PRISMA reporting guidelines.

### Search strategy

We aimed to find both published and unpublished studies. The databases searched included PubMed (NIH), Embase (Embase.com), Web of Science (Clarivate), CINAHL (ebsco.com), and Scopus (Elsevier). Sources of unpublished studies and gray literature searched included Cochrane Central Register of Controlled Trials (Wiley), National Health Service Economic Evaluation Database (York, Centre for Reviews and Dissemination), ClinicalTrails.gov, and Cost-Effectiveness Analysis Registry. The search strategies for all searched databases and information sources are listed in Supplementary Table 1. The search strategy, including all identified keywords and index terms, was adapted for each included database and/or information source. A hand search was completed, which included a review of the included articles’ references and exploration of related articles identified.

Studies published in English from 1 January 2011 to 8 November 2021 were considered for inclusion. The limits of publication dates were based on historically impactful federal decisions. Throughout the previous decade, technologies associated with RPM development were promoted with healthcare reforms and federal legislation. The National Broadband Plan by the Federal Communications Commission in 2010 facilitated medical technology advancement and the development of technology-based health services (17). As such, the date limit of this review will be set starting from 2011.

### Eligibility criteria

A PICO framework (Population, Intervention, Comparator, Outcomes) was used for study selection. Articles were eligible for inclusion if they involved a chronic CVD patient population in the US, comparing RPM, or similar health delivery models, with usual care in terms of costs or in conjunction with other health benefit outcomes. This review considered studies evaluating the costs from all time horizons, for example, short-term and long-term, and all perspectives, for example, payer, provider, and healthcare sectors (18). Full economic evaluation studies, such as cost-effectiveness analysis (CEA), cost-utility analysis (CUA), and cost–benefit analysis (CBA), as well as partial economic evaluation studies, such as cost analysis, cost-description studies, and cost-outcome descriptions, were considered for inclusion in the review (15;19).

### Study selection

Following the search, all identified citations were collated and uploaded into EndNote V20 (Clarivate Analytics, PA, USA), and duplicates were removed. Titles and abstracts were screened by two independent reviewers (YZ and MTP) for assessment against the inclusion criteria. The full text of selected studies was assessed in detail against the inclusion criteria by two independent reviewers (YZ and MTP). Disagreements during any selection stage were resolved through discussion between all study members. All screenings were completed through Rayyan (20), a free online systematic review data management software.

### Assessment of methodological quality

Eligible studies were critically appraised by two independent reviewers (YZ and MTP) at the study level for methodological quality following the detailed checklist for assessing economic evaluation from Drummond et al. (19) and summarized using standardized critical appraisal instruments from the Joanna Briggs Institute for Economic Evaluation (15). Any disagreements that arose between the reviewers were resolved through discussion or with all study members.

### Data extraction and analysis

Data were extracted from studies included in the review by two independent reviewers (YZ and MTP) using a modified JBI data extraction tool for economic evaluation (15), to consider key elements of both partial and full economic evaluations. The data extracted include specific details about the participants, study design, interventions, comparators, study perspectives, time horizons, analysis type, clinical effectiveness, and costs and cost-effectiveness outcomes of significance to the review objective (16). If an article reported results from multiple studies, we extracted data for all eligible studies and analyzed them separately. If a study reported multiple outcomes, we extracted the results for a broader range of costs, a more commonly used effectiveness outcome, and a longer time horizon. In conducting economic analysis, it is imperative to have a clear study perspective (19). Two reviewers inferred the study perspective and the year of cost data, if it was not explicitly stated in the article. Any disagreements that arose between the reviewers were resolved through discussion.

All extracted cost data were converted to 2021 US dollars ($) using a web-based tool developed by the Campbell and Cochrane Economics Methods Group and the Evidence for Policy and Practice Information and Coordinating Centre (EPPI-Centre) (21). Results of included studies were synthesized by economic analysis types and were interpreted considering study perspectives, intervention, health outcomes, and time horizons. Data from full economic evaluations are summarized using JBI Dominance Ranking Matrix (15), which ranks studies by cost and health benefit and synthesizes them into three implication categories: reject intervention, unclear, and favor intervention.

## Results

### Study inclusion

**Figure 1.**
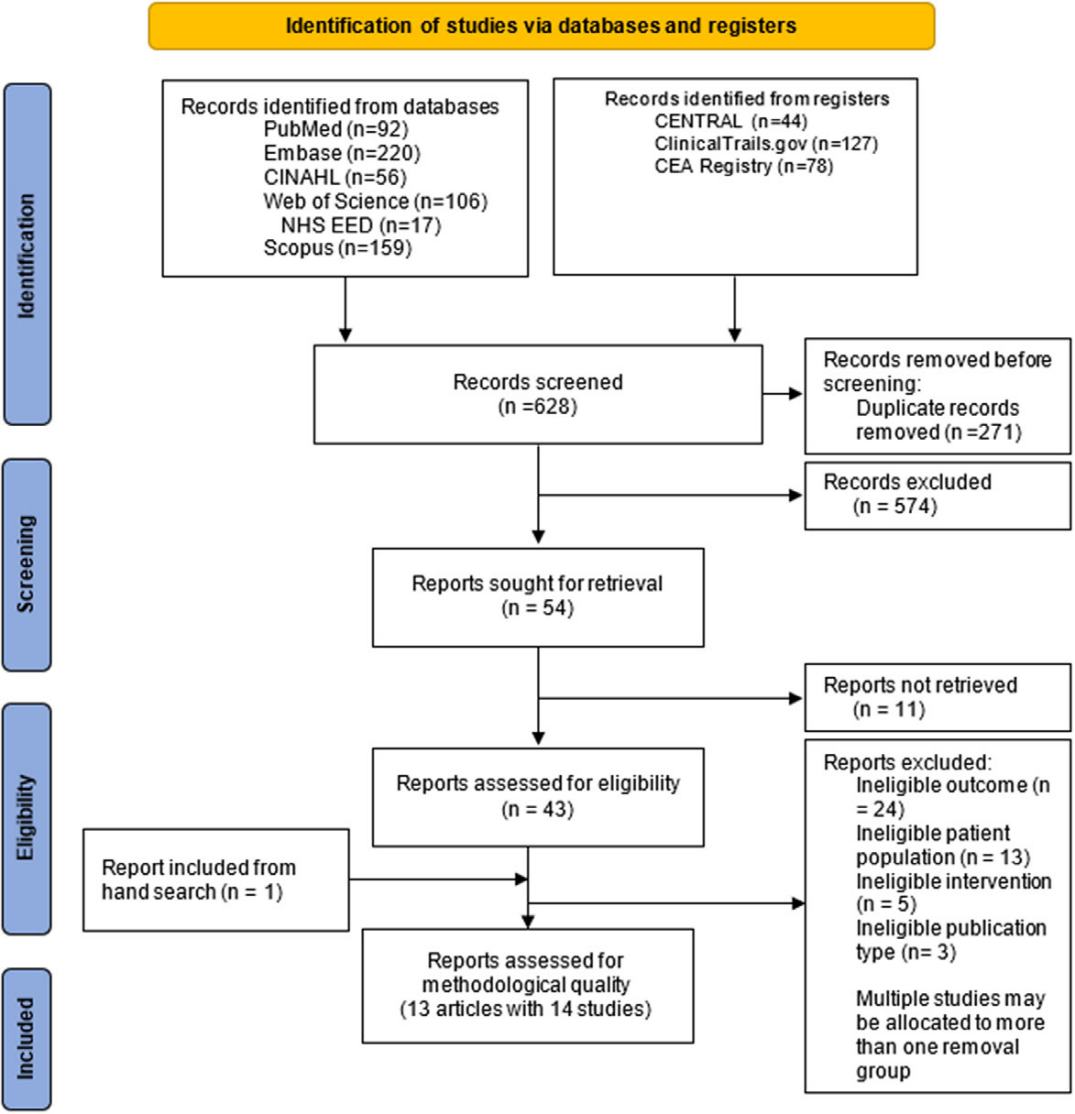
PRISMA flowchart describing the result of the search and selection process.

As shown in the PRISMA flowchart (Figure 1), 899 records were identified from databases and registries. After the exclusion of 271 duplicates, 628 titles and abstracts were screened. Among the fifty-four potentially relevant studies, eleven clinical trial registrations did not have final reports after contacting principal investigators. Forty-three full-text reviews revealed thirty-one papers excluded for ineligible publication type, patient populations, interventions, or outcomes. One additional article was included through hand searching. One article reported two eligible independent studies, so we consider them as separate studies in the analysis (22). Consequently, thirteen articles, with fourteen studies, were included in the final review for data extraction and synthesis. Among them, eight conducted partial economic evaluations, in which data about treatment costs and effectiveness were presented without an integrated analysis of cost and effectiveness (22–28). The remaining six studies conducted full economic evaluations, three contained CEA (29–31), two contained CUA (32;33), and one contained CBA and return on investment analysis (28).

### Methodological quality

Overall, seventy-three percent of the quality criteria were met. Among all the included articles, full economic evaluations had higher methodological quality than partial economic evaluations; on average, full economic evaluations met eighty-nine percent of the quality criteria, while partial economic evaluations met sixty-one percent. Two partial and four full economic evaluations had a well-defined question along with a study perspective clearly stated (24;27;29–32). Four studies did not have a clear study perspective; therefore, it was unclear if any relevant costs or outcomes were omitted (25;26;33;34). All included studies compared clinical effectiveness between the intervention and comparator groups with costs and effectiveness data measured accurately and credibly. Four full economic evaluations adjusted costs and outcomes for differential timing (28;30;32;33). The detailed methodological quality assessment is provided in Supplementary Table 2.

### Characteristics of included studies

Table 1 provides an overview of the study characteristics and primary results. The clinical efficacy data in the included studies were from randomized clinical trials (RCTs) (22–25;27;29;30;32–34) and quasi-experimental studies (22;26;31;35). Generally, this systematic review included a total of 3,915 actual patients, with 2,304 in RPM intervention groups and 200,000 simulated patients with 100,000 in the RPM intervention group.

**Table 1.** Characteristics of included studies

Study	Location, year of cost data, clinical study type	Intervention	Sample size	Model type	Analysis type	Time horizon (years)	Raw costs
*Partial economic evaluation*							
Blum et al. (23)	Baltimore/Washington DC metropolitan area, ~2006, RCT	RPM	IG: 104 CG: 102	Trial based	Cost analysis and cost-outcome description	4	Total Medicare payment per subject, mean (SD), IG vs CG: $64,788 (100,452) vs $40,480 (58,572)
Maciejewski et al. (24)	North Carolina, 2010, RCT	IG1: RPM + medication management IG2: RPM + behavioral management IG3: RPM + behavioral and medication management	IG1: 149 IG2: 148 IG3: 147 CG: 147	Trial based	Cost analysis and cost-outcome description	3	Total expenditure, mean (95% CI): IG1 – CG: -$977 (−2,715 to 761) IG2 – CG: $3,237 (−2,837 to 9,312) IG3 – CG: $309 (−1,643 to 2,262)
Nouryan et al. (25)	US, ~2017, RCT	RPM	IG: 42 CG: 47	Trial based	Cost analysis and cost-outcome description	0.5	All-cause charges to Medicare, mean (SD), IG vs CG: $38,990 (69,031) vs $50,943 (98,519)
Pekmezaris et al. (22)	New York metropolitan area, ~ 2009, RCT and QE	RPM	RCT: IG 83, CG 85 QE: IG 80, CG 79	Trial based	Cost analysis and cost-outcome description	2	Cost to Medicare, mean (SD): RCT: IG $7,267 (13, 355), CG $8,048 (15, 118) QE: IG $3,555 (7, 936), CG $2,532 (7, 689)
Riley et al. (26)	Arizona, including underserved rural communities and native American reservations, ~2014, QE	RPM	IG: 45 CG: 45	Trial based	Cost analysis and cost-outcome description	1	Total hospital charges, mean (SD): IG: pre $138,600 (136,741), post $44,674 (717,509) CG: pre $92,930 (102,947), post $39,732 (71,750)
Wang et al. (27)	North Carolina, ~2009, RCT	IG1: RPM + medication management IG2: RM + behavioral management IG3: RPM + behavioral and medication management	IG1: 149 IG2: 148 IG3: 147 CG: 147	Trial based	Cost analysis and cost-outcome description	1.5	Total costs: IG1 – CG, mean (95% CI): $2,125 (−2,068 to 8,376) IG2 – CG, mean (95% CI): $2,113 (−1,820 to 6,361) IG3 – CG, mean (95% CI): $681 (−3,061 to 5,528)
White-Williams et al. (35)	US, ~2010, QE	RPM	IG: 235 CG: 91	Trial based	Cost analysis and cost-outcome description	1	Average cost per patient, IG vs CG: $415 vs $619
*Full economic evaluation*							
Dehmer et al. (29)	Minneapolis-St. Paul metropolitan area, ~2017, RCT	RPM + medication management	IG: 148 CG: 150	Trial based	CEA	1	Intervention costs, mean (95% CI): $1,350 (615 to 2,080) ICER (95% CI): $7,337 (2, 278, 26, 329) per person achieving BP control
Fishman et al. (30)	Washington, 2009, RCT	IG1: RPM IG2: RPM + medication management	IG1: 259 IG2: 261 CG: 258	Trial based	CEA	1	Total program costs per patient, mean (range): IG1: $67 (54 to 81) IG2: $400 (263 to 566) CG: $11 (8 to 13) ICER: IG1 vs CG: NS IG2 vs IG1, mean (95% CI): $17 (15 to 18) achieving 1% improvement in the number of patients with BP control
Margolis et al. (34)	Minneapolis-St. Paul metropolitan area, 2017, RCT	RPM + medication management	IG: 228 CG: 222	Model based	CBA and ROI	5	IG Intervention cost: $1,511 Benefit–cost ratio: 2.19 ROI: 119%
Martinson et al. (32)	US, 2014, RCT	RPM with implantable sensor	IG: 100,000 CG: 100,000	Model based	CUA	3–7	Cumulative average cost over 5-year, IG vs CG: $140,966 vs $133,681 ICER over 5-year: $18,515 per QALY
Schmier et al. (33)	US, 2016, RCT	RPM with implantable sensor	IG: 270 CG: 280	Model based	CUA	5	Total costs, mean (SD), IG vs CG: $188,880 vs $162,772 ICER: $44,832 per QALY
Willams et al. (31)	US, ~2013, QE	RPM	IG: 105 CG:105	Trial based	CEA	2	Agency cost, mean (SD), IG vs CG: $982 (404) vs $522 (320) Average cost-outcomes ratio $153

BP, blood pressure; CBA, cost–benefit analysis; CEA, cost-effectiveness analysis; CG, control group; CUA, cost-utility analysis; ICER, incremental cost-effectiveness ratio; IG, intervention group; NS, not significant; ~ indicates data inferred by reviewers; RCT, randomized clinical trial; RPM, remote patient monitoring; ROI, return on investment; QE, quasi-experimental study.

Though RPM served as the basic concept of all interventions in this review, studies may have additive interventional medical services. Five studies, two partial and three full economic evaluations, had an intervention group of RPM with medication management (24;27;29;30;34). Two studies conducting partial economic evaluations based on the same RCT had intervention groups of RPM with behavioral management and RPM with behavioral and medication combined management (24;27). In addition, two studies were based on the same RCT of an implantable wireless pulmonary artery pressure remote monitor (32;33).

The economic impact of RPM for a time horizon of half to two years was assessed in nine studies (22;25–27;29–31;35). Two partial and three full economic evaluations were conducted for a longer time horizon between two and seven years, of which three full economic evaluations were model based, consisting of two CUAs and one CBA (32–34). Moreover, the included studies were of varying perspectives, including nine studies from the payer (22;23;25;26;28;32;33;35), two studies from the provider (30;31), two studies from the Veteran Affairs (VA) healthcare system (24;27), and one study from the healthcare sector perspective (29). The relevant types of costs used in each study with its study perspective are summarized in Table 2. Certain types of costs were excluded in the cost aggregates if their difference was not significant between RPM and comparator groups.

**Table 2.** Reported cost components (*N* = 14)

		Partial economic evaluation							Full economic evaluation					
	Blum et al. (23)	Maciejewski et al. (24)	Nouryan et al. (25)	Pekmezaris et al. (22) (RCT)	Pekmezaris et al. (22) (QE)	Riley et al. (26)	Wang et al. (27)	White-Williams et al. (35)	Dehmer et al. (29)	Fishman et al. (30)	Margolis et al. (34)	Martinson et al. (32)	Schmier et al. (33)	Willams et al. (31)
Perspective	~Payer	VA healthcare	~Payer	~Payer	~Payer	~Payer	VA healthcare	Payer	Healthcare sector	Provider	~Payer	Payer	~Payer	Provider
Program costs														
Equipment and supplies	—	—	—	—	—	—	✓	—	—	✓	—	—	—	✓
Personnel	—	—	—	—	—	—	✓	—	—	✓	—	—	—	✓
Marketing	—	—	—	—	—	—	—	—	—	—	—	—	—	✓
Overhead costs	—	—	—	—	—	—	—	—	—	✓	—	—	—	—
Medical costs														
Outpatient	—	✓	✓	—	—	—	✓	—	✓	—	✓	✓	✓	—
Inpatient	✓	✓	✓	✓	✓	✓	✓	✓	✓	—	✓	✓	✓	—
ED	✓	—	✓	✓	✓	✓	—	✓	✓	—	✓	—	—	—
Medication	—	—	—	—	—	—	✓	—	✓	—	✓	✓	—	—
Critical care service	—	—	—	—	—	—	—	—	✓	—	—	—	—	—
Evaluation/management/observation service	—	—	—	—	—	—	—	—	✓	—	—	—	—	—
Laboratory test	—	—	—	—	—	—	—	—	✓	—	—	—	—	—
Procedure	—	—	—	—	—	—	—	—	—	—	✓	—	✓	—
Radiology service	—	—	—	—	—	—	—	—	✓	—	—	—	—	—
Complication	—	—	—	—	—	—	—	—	—	—	—	✓	✓	—
Monitoring device	—	—	—	—	—	—	—	—	—	—	—	✓	✓	—

### Program costs

Three studies reported program costs such as costs of equipment and medical supplies, personnel, marketing, and overhead (27;30;31). The equipment and supplies may consist of laptop computers and accessories, home BP monitors, and telemedicine devices for data transmission and their warranty, batteries for BP monitors, and medication containers. The personnel costs were calculated based on personnel time and relevant hourly wage, along with additional benefits. Personnel time contained nurse time spent receiving training, educating patients, implementing programs, calling patients and preparing for calls, and physician time spent reviewing medical charts and consulting with nurses. For RPM with medication management, costs were considered for pharmacist time spent receiving training, developing protocols, and providing services, such as reviewing patient progress and medication regimens, monitoring potential adverse events, and meeting with physicians.

Table 3 displays the cost and cost-effectiveness of each study inflated to 2021 US Dollars ($) by health benefit. Among the three studies that reported program costs, one study from the VA healthcare perspective found no difference in program costs and treatment efficacy between RPM and the usual care group (27). In comparison, the two studies from the provider perspective reported higher costs for the RPM-only group with no difference in treatment efficacy (30;31).

**Table 3.** Cost and cost-effectiveness results by effectiveness outcomes in 2021 US dollars (*N* = 14)

	Intervention group cost	Control group cost	Cost-effectiveness	Results
*Hospitalization*				
Pekmezaris et al. (22), RCT	$8,955	$9,918	—	Equal cost, equal effectiveness
Pekmezaris et al. (22), QE	$4,381	$3,120	—	Equal cost, equal effectiveness
Riley et al. (26)	Pre $156,506, Post $50,446	Pre $104,936, Post $44,865	—	Equal cost, equal effectiveness
White-Williams et al. (35)	$506	$755	—	Equal cost, equal effectiveness
Williams et al. (31)	$1,129	$600	Average cost-outcomes ratio $176	Higher cost, equal effectiveness
*BP control*				
Maciejewski et al. (24)	IG1: -$1,190 IG2: $3,943 IG3: $376	RG	—	IG1/IG2/IG3 vs CG: equal cost, better effectiveness
Wang et al. (27)	IG1: $2,614 IG2: $2,599 IG3: $838	RG	—	IG1/IG2/IG3 vs CG: equal cost, equal effectiveness
Dehmer et al. (29)	$1,458	RG	ICER: $7,924 per person achieving BP control	Higher cost, better effectiveness
Fishman et al. (30)	IG1: $82 IG2: $492	$14	IG1 vs CG: NS IG2 vs IG1: ICER $21 achieving 1% improvement in the number of patients with BP control	IG1 vs CG: higher cost, equal effectiveness IG2 vs IG1: higher cost, better effectiveness
*Quality of life*				
Blum et al. (23)	$84,217	$52,619	—	Equal cost, equal effectiveness
Nouryan et al. (25)	$42,287	$55,250	—	Equal cost, better effectiveness
*QALYs*				
Martinson et al. (32)	$159,325	$151,060	ICER: $20,922 per QALY	Higher cost, better effectiveness
Schmier et al. (33)	$209,656	$180,677	ICER: $49,764 per QALY	Higher cost, better effectiveness
*CVD events*				
Margolis et al. (34)	$1,632	RG	ROI: 119% Benefit–cost ratio: 2.19	Lower cost, better effectiveness

BP, blood pressure; CG, control group; CVD, cardiovascular disease; ICER, incremental cost-effectiveness ratio; IG, intervention group; QALY, quality-adjusted life year; NS, not significant; RG, reference group.

### Medical costs

Inpatient, emergency department, and outpatient costs were the most commonly considered components among included studies. Medication costs were considered in four studies (27–29;32). Other medical costs may include critical care, laboratory test, procedure, radiology, evaluation/management/observation service, complication, and monitoring service. As opposed to the two studies from the provider perspective that considered monitoring as a program cost (30;31), two studies using the implantable device considered it as a monthly cost to the payer (32;33). Among studies that reported medical costs for the RPM-only intervention, seven partial economic evaluation studies reported no significant difference in costs between groups (22–27;35), and two full economic evaluation studies reported that the RPM group costs more than the usual care group (details can be found in Table 3) (32;33).

### Cost-effectiveness

When considering costs and health outcomes jointly, the included partial economic evaluations tend to favor the RPM intervention. Maciejewsk et al. (24) and Nouryan et al. (25) reported that, with the same costs, patients receiving RPMs had better BP control and improved quality of life than usual care patients. Included partial economic evaluations reported equal costs and efficacy between RPM and usual care groups for hospitalization-related outcomes, such as the number of readmission and readmission rate. In assessing costs of BP control, two partial economic evaluations based on the same RCT found no difference in associated costs among groups (24;27); however, the one with a longer time horizon identified all RPM groups as having better BP control than the usual care group (21). The two partial economic evaluations that evaluated the quality of care had differing results (23;25).

Moreover, the results of full economic evaluations are summarized using JBI Dominance Ranking Matrix. Table 4 shows the synthesized results from two CEA and two CUA studies that compared the RPM-only intervention with the usual care. The RPM-only intervention was rejected in two CEA studies from the provider perspective, due to higher costs and similar efficacy for the RPM group relative to the usual care group. Both CUA studies reported that the RPM group had higher costs and Quality-Adjusted Life-Years (QALYs) than usual care and showed unclear implications; however, at a conservative threshold of $50,000 per QALY, the CUA studies suggest that RPM relative to usual care is a cost-effective tool for CVD management (32;33). Additionally, Supplementary Table 3 shows the synthesized results for RPM with medication management intervention. Two CEA studies found that RPM with medication management led to higher costs and better BP control (29;30), which were cost-effective at the willingness-to-pay of $150,000 per QALY, and a CBA study found that avoiding CVD events led to cost savings (34). Supplementary Table 4 shows the synthesized results for studies with a time horizon over three years. Among three full economic evaluations with at least a three-year time horizon, the CBAs favored RPM with medication management, and both CUAs favored RPM at the $50,000 per QALY threshold (32;33).

**Table 4.** JBI dominance ranking matrix of all included full economic evaluations for RPM-only (*n* = 4)

Cost	Health benefit	Implication for decision makers	No. of studies
+	−	Reject intervention	
0	−	Reject intervention	
+	0	Reject intervention	2 (Fishman et al. (30); Williams et al. (31))
−	−	Unclear: judgment required on whether intervention is preferable considering incremental cost-effectiveness measures and priorities/willingness to pay	
0	0	Unclear: judgment required on whether intervention is preferable considering incremental cost-effectiveness measures and priorities/willingness to pay	
+	+	Unclear: judgment required on whether intervention is preferable considering incremental cost-effectiveness measures and priorities/willingness to pay	2 (Martinson et al. (32); Schmier et al. (33))
−	0	Favor intervention	
0	+	Favor intervention	
−	+	Favor intervention	

## Discussion

This systematic review aimed to assess the costs and cost-effectiveness of RPM for CVD management in the US. Our review included fourteen studies reported in thirteen articles comparing RPM-based interventions with usual care in terms of cost and cost-effectiveness for CVD management. Partial economic evaluations found similar costs between RPM and usual care groups, while full economic evaluations, using different outcomes and analysis methods, suggested that RPM and RPM with medication management are cost-effective in terms of QALYs, BP control, and reducing the incidence of CVD events, especially in the long run. To fully understand the economic impact, it is essential to consider long-term costs and clinical outcomes.

The present study is the first systematic review of the economic impact of RPM for CVD management, focusing on the US healthcare system. A previous systematic review involved thirty-four studies conducted in twelve countries for multiple chronic diseases (36). Their results revealed that RPM is a highly cost-effective intervention for hypertension and prevents high-cost health events in the long run from a wide variety of perspectives, which is consistent with our findings of studies from payer and healthcare perspectives (36). However, our review includes a different set of studies, including partial and full economic evaluations, invasive and non-invasive RPM, and focused on CVD management in the US context. When examining the cost-effectiveness of a program in the US healthcare system context, it is important to consider the healthcare provider perspective, which informs policymakers regarding payer–provider partnerships. Program costs, referring to costs incurred at the administrative level (37), are an essential component of RPM-based interventions, especially for studies that include the provider perspective. Two included full economic evaluations revealed higher program costs of RPM compared to usual care with additional monitor and data transmission devices as well as nurse time and pharmacist time for training, service, and communication with patients. In addition, medication costs constitute an important portion of assessing the cost-effectiveness of health interventions, which is a cost borne at least partly by patients in the US, depending on the payer type. RPM with medication management can enhance medication adherence and ultimately reduce healthcare costs (38); therefore, it is important to consider medication costs in economic analysis. We recommend that future economic studies of RPM should be conducted rigorously with a broader perspective, such as societal perspective to include the provider perspective, considering program costs, and the patient perspective, considering medication costs (39).

Nevertheless, the study of Riley et al. (26) was the only one with specified locations that included underserved rural communities and Native American reservations. Though the clinical efficacy of RPM has been addressed in rural areas and low-income populations (40), future evidence of RPM cost-effectiveness in underserved populations is needed.

There are some limitations within our systematic review. Although fourteen studies were included in the review, eight were partial economic evaluations, providing lower quality evidence. The heterogeneity of patient populations, varying study perspectives, different health benefit outcomes, and the small number of articles included make it challenging to draw conclusions in each category and limit the generalizability of these findings.

## Conclusions

This review summarizes current evidence of the economic impact of RPM on CVD management in the US. The findings suggest that RPM-based healthcare services can be more cost-effective than usual care from the payer perspective for CVD management in terms of QALYs, BP control, and fewer CVD events. This result can seem encouraging for third-party payers. Given the current evidence of clinical effectiveness, future efforts should seek to investigate the economic sustainability of RPM for CVD management, considering broader and varying perspectives with a longer-term view.

## Supplementary material

For supplementary material accompanying this paper visit https://doi.org/10.1017/S0266462323000156. click here to view supplementary material

## References

[1] World Health Organization. Cardiovascular diseases (CVDs). World Health Organization [cited 2022 Apr 10]. 2021. Available from: https://www.who.int/en/news-room/fact-sheets/detail/cardiovascular-diseases-(cvds).

[2] Virani SS, Alonso A, Aparicio HJ, et al. Heart disease and stroke statistics - 2021 update. Circulation. 2021;143(8):e254–e743. 33501848 10.1161/CIR.0000000000000950 PMC13036842

[3] Centers for Disease Control and Prevention. Health and Economic Costs of Chronic Diseases. Centers for Disease Control and Prevention. [cited 2021 Aug11]. 2021. Available from: https://www.cdc.gov/chronicdisease/about/costs/index.htm.

[4] American Heart Association. Using remote patient monitoring technologies for better cardiovascular disease outcomes: guidance. 2019. https://www.heart.org/-/media/files/about-us/policy-research/policy-positions/clinical-care/remote-patient-monitoring-guidance-2019.pdf

[5] Farias FAC, Dagostini CM, Bicca YA, Falavigna VF, Falavigna A. Remote patient monitoring: A systematic review. Telemed J E Health. 2020;26(5):576–583. 31314689 10.1089/tmj.2019.0066

[6] Davis TC, Hoover KW, Keller S, Replogle WH. Mississippi diabetes telehealth network: A collaborative approach to chronic care management. Telemed e-Health. 2019;26(2):184–189. 10.1089/tmj.2018.0334 30822265

[7] Nakamura N, Koga T, Iseki H. A meta-analysis of remote patient monitoring for chronic heart failure patients. J Telemed Telecare. 2014;20(1):11–17. 24352899 10.1177/1357633X13517352

[8] Parthiban N, Esterman A, Mahajan R, et al. Remote monitoring of implantable cardioverter-defibrillators: A systematic review and meta-analysis of clinical outcomes. J Am Coll Cardiol. 2015;65(24):2591–2600. 25983009 10.1016/j.jacc.2015.04.029

[9] Bashi N, Karunanithi M, Fatehi F, Ding H, Walters D. Remote monitoring of patients with heart failure: An overview of systematic reviews. J Med Internet Res. 2017;19(1):e18. 28108430 10.2196/jmir.6571 PMC5291866

[10] Taylor ML, Thomas EE, Snoswell CL, Smith AC, Caffery LJ. Does remote patient monitoring reduce acute care use? A systematic review. BMJ Open. 2021;11(3):e040232. 10.1136/bmjopen-2020-040232 PMC7929874 33653740

[11] Thomas EE, Taylor ML, Banbury A, et al. Factors influencing the effectiveness of remote patient monitoring interventions: A realist review. BMJ Open. 2021;11(8):e051844. 10.1136/bmjopen-2021-051844 PMC8388293 34433611

[12] Summary of policies in the calendar year (CY). Medicare physician fee schedule (MPFS) final rule, Telehealth originating site facility fee payment amount and Telehealth services list, and CT modifier reduction list [database on the Internet]. 2017. 2018. Available from: https://www.cms.gov/Outreach-and-Education/Medicare-Learning-Network-MLN/MLNMattersArticles/downloads/MM10393.pdf.

[13] Office of the Federal Register, National Archives and Records Administration. 86 FR 64996 - Medicare Program; CY 2022 Payment Policies Under the Physician Fee Schedule and Other Changes to Part B Payment Policies; Medicare Shared Savings Program Requirements; Provider Enrollment Regulation Updates; and Provider and Supplier Prepayment and Post-Payment Medical Review Requirements. Office of the Federal Register, National Archives and Records Administration. 2021.

[14] Benjamin EJ, Virani SS, Callaway CW, et al. Heart disease and stroke statistics—2018 update: A report from the American heart association. Circulation. 2018;137(12):e67–e492. 29386200 10.1161/CIR.0000000000000558

[15] Gomersall J, Jadotte Y, Xue Y, et al. Chapter 6: Systematic reviews of economic evidence. [cited 2021 Aug 11]. In: JBI manual for evidence synthesis [Internet]. Adelaide: JBI. [cited 2021 Aug 11]. 2020. Available from: https://synthesismanual.jbi.global.

[16] Zhang Y, Peña MT, Fletcher LM, Swint JM, Reneker JC. Cost of remote patient monitoring for cardiovascular disease: A systematic review protocol. JBI Evid Synth. 2022;20:1585–1592. 35142743 10.11124/JBIES-21-00322

[17] Gruessner V. The History of Remote Monitoring, Telemedicine Technology. mHealthIntelligence; [cited 2021 Aug 23]. 2015. Available from: https://mhealthintelligence.com/news/the-history-of-remote-monitoring-telemedicine-technology.

[18] Drummond M, Jefferson T. Guidelines for authors and peer reviewers of economic submissions to the BMJ. The BMJ Economic Evaluation Working Party. BMJ. 1996;313:275. 8704542 10.1136/bmj.313.7052.275 PMC2351717

[19] Drummond MF, Sculpher MJ, Claxton K, Stoddart GL, Torrance GW. Methods for the economic evaluation of health care programmes. Oxford: Oxford University Press; 2015.

[20] Ouzzani M, Hammady H, Fedorowicz Z, Elmagarmid A. Rayyan—A web and mobile app for systematic reviews. Syst Rev. 2016;5:210. 27919275 10.1186/s13643-016-0384-4 PMC5139140

[21] CCEMG - EPPI-Centre. CCEMG – EPPI-Centre Cost Converter. 2019. Available from: https://eppi.ioe.ac.uk/costconversion/default.aspx.

[22] Pekmezaris R, Mitzner I, Pecinka KR, et al. The impact of remote patient monitoring (telehealth) upon medicare beneficiaries with heart failure. Telemed J E Health. 2012;18(2):101–108. 22283360 10.1089/tmj.2011.0095

[23] Blum K, Gottlieb SS. The effect of a randomized trial of home telemonitoring on medical costs, 30-day readmissions, mortality, and health-related quality of life in a cohort of community-dwelling heart failure patients. J Card Fail. 2014;20(7):513–521. 24769270 10.1016/j.cardfail.2014.04.016

[24] Maciejewski ML, Bosworth HB, Olsen MK, et al. Do the benefits of participation in a hypertension self-management trial persist after patients resume usual care?. Circ Cardiovasc Qual Outcomes. 2014;7(2):269–275. 24619321 10.1161/CIRCOUTCOMES.113.000309

[25] Nouryan CN, Morahan S, Pecinka K, et al. Home telemonitoring of community-dwelling heart failure patients after home care discharge. Telemed J E Health. 2019;25(6):447–454. 30036166 10.1089/tmj.2018.0099

[26] Riley WT, Keberlein P, Sorenson G, et al. Program evaluation of remote heart failure monitoring: Healthcare utilization analysis in a rural regional medical center. Telemed J E Health. 2015;21(3):157–162. 25025239 10.1089/tmj.2014.0093 PMC4365431

[27] Wang V, Smith VA, Bosworth HB, et al. Economic evaluation of telephone self-management interventions for blood pressure control. Am Heart J. 2012;163(6):980–986. 22709750 10.1016/j.ahj.2012.03.016

[28] Margolis KL, Asche SE, Bergdall AR, et al. Effect of home blood pressure telemonitoring and pharmacist management on blood pressure control: A cluster randomized clinical trial. JAMA. 2013;310(1):46–56. 23821088 10.1001/jama.2013.6549 PMC4311883

[29] Dehmer SP, Maciosek MV, Trower NK, et al. Economic evaluation of the home blood pressure telemonitoring and pharmacist case management to control hypertension (hyperlink) Trial. J Am Coll Clin Pharm. 2018;1(1):21–30. 30320302 10.1002/jac5.1001 PMC6181443

[30] Fishman PA, Cook AJ, Anderson ML, et al. Improving BP control through electronic communications: An economic evaluation. Am J Manag Care. 2013;19(9):709–716. 24304254 PMC3938103

[31] Williams C, Wan TTH. A cost analysis of remote monitoring in a heart failure program. Home Health Care Serv Q. 2016;35(3–4):112–122. 27552654 10.1080/01621424.2016.1227009

[32] Martinson M, Bharmi R, Dalal N, Abraham WT, Adamson PB. Pulmonary artery pressure-guided heart failure management: US cost-effectiveness analyses using the results of the CHAMPION clinical trial. Eur J Heart Fail. 2017;19(5):652–660. 27647784 10.1002/ejhf.642 PMC5434920

[33] Schmier JK, Ong KL, Fonarow GC. Cost-effectiveness of remote cardiac monitoring with the CardioMEMS heart failure system. Clin Cardiol. 2017;40(7):430–436. 28272808 10.1002/clc.22696 PMC6490396

[34] Margolis KL, Dehmer SP, Sperl-Hillen J, et al. Cardiovascular events and costs with home blood pressure telemonitoring and pharmacist management for uncontrolled hypertension. Hypertension. 2020;76(4):1097–1103. 32862713 10.1161/HYPERTENSIONAHA.120.15492 PMC7484110

[35] White-Williams C, Unruh L, Ward K. Hospital utilization after a telemonitoring program: A pilot study. Home Health Care Serv Q. 2015;34(1):1–13. 25517540 10.1080/01621424.2014.995256

[36] De Guzman KR, Snoswell CL, Taylor ML, Gray LC, Caffery LJ. Economic evaluations of remote patient monitoring for chronic disease: A systematic review. Value Health. 2022 5(6):897–913. 10.1016/j.jval.2021.12.001 35667780

[37] Johns B, Baltussen R, Hutubessy R. Programme costs in the economic evaluation of health interventions. Cost Eff. Resour. Alloc. 2003;1(1):1. 12773220 10.1186/1478-7547-1-1 PMC156020

[38] Simon ST, Kini V, Levy AE, Ho PM. Medication adherence in cardiovascular medicine. BMJ. 2021;374:n1493. 34380627 10.1136/bmj.n1493

[39] Khera R, Valero-Elizondo J, Das SR, et al. Cost-related medication nonadherence in adults with atherosclerotic cardiovascular disease in the United States, 2013 to 2017. Circulation. 2019;140(25):2067–2075. 31760784 10.1161/CIRCULATIONAHA.119.041974

[40] Clark D, 3rd, Woods J, Zhang Y, et al. Home blood pressure telemonitoring with remote hypertension management in a rural and low-income population. Hypertension. 2021;78(6):1927–1929. 34757773 10.1161/HYPERTENSIONAHA.121.18153 PMC8589114

