# Supplementary material for: Economic evaluation and costs of remote patient monitoring for cardiovascular disease in the United States: a systematic review
Source: Int J Technol Assess Health Care. 2023 Apr 28;39(1):e25. doi: 10.1017/S0266462323000156 (PMC11574531; doi:10.1017/S0266462323000156)
Supplement: Supplementary file 1 [file S0266462323000156sup001.docx]

**Supplemental Material**

**Supplementary Table 1**. Search strategies for all databases and information sources.

**PUBMED (NIH)**

1. (Economic evaluation[tiab] OR Cost-effectiv* analysis[tiab] OR cost utility analysis[tiab] OR cost benefit analysis[tiab] OR cost minimization analysis[tiab] OR Cost of illness[tiab] OR Costs and cost analysis[tiab])
2. Costs and cost analysis[mesh]
3. (Healthcare cost*[tiab] OR Health care cost*[tiab] OR Economic benefit*[tiab] OR Spending[tiab] OR Expenditure[tiab] OR Cost minimiz*[tiab] OR Economics[tiab])
4. #1 OR #2 OR #3
5. (remote patient monitoring [tiab] OR remote monitoring [tiab] OR remote care[tiab] OR remote consultation*[tiab])
6. (Telemedicine[tiab] OR Telecare[tiab] OR Telehealth[tiab] OR Telenursing[tiab] OR Telemonitoring[tiab] OR Tele monitoring[tiab] OR Telemetric[tiab] OR Telehomecare[tiab] OR Tele homecare[tiab] OR Telemanagement[tiab] OR Tele management[tiab] OR Telerehabilitation[tiab] OR tele rehabilitation[tiab] OR telesurveillance[tiab] OR tele surveillance[tiab])
7. Telemedicine[mesh]
8. (Ehealth[tiab] OR e-Health[tiab] OR mhealth[tiab] OR m-Health[tiab])
9. #5 OR #6 OR #7 OR #8
10. (home care services[tiab] OR homecare services[tiab] OR homecare[tiab] OR home care[tiab] OR home[tiab] OR telehomecare[tiab] OR tele homecare[tiab] OR home telehealth[tiab])
11. home care services[mesh]
12. #10 OR #11
13. (heart failure[mesh] OR heart failure[tiab] OR congestive heart failure[tiab] OR cardiac failure[tiab])
14. (hypertension[mesh] OR hypertension[tiab] OR high blood pressure[tiab])
15. (Heart diseases[Mesh:NoExp] OR heart diseas*[tiab] OR cardiac diseas*[tiab])
16. (heart valve diseases[mesh] OR heart valve diseas*[tiab] OR heart valve disorder*[tiab] OR valvular heart diseas* OR pericarditis[mesh] OR pericarditis[tiab] OR endocarditis[mesh] OR endocarditis[tiab] OR myocarditis[mesh] OR myocarditis[tiab] OR cardiomyopath*[tiab] OR cardiomyopathies[mesh] OR coronary atherosclerosis[tiab] OR coronary artery atherosclerosis[tiab] OR coronary artery disease[mesh] OR coronary artery diseas*[tiab] OR pulmonary heart disease[mesh] OR pulmonary heart diseas*[tiab] OR cor pulmonale[tiab] OR pulmonary cardiac diseas*[tiab] OR conduction disorder*[tiab] OR cardiac conduction system disease[mesh] OR cardiac conduction system diseas*[tiab] OR heart muscle conduction disturbance[tiab] OR cardiac dysrhythmia*[tiab] OR arrhythmias, cardiac[mesh] OR cardiac arrhythmia*[tiab] OR atrial fibrillation[tiab] OR ventricular fibrillation[tiab] OR hypertension with heart diseas*[tiab])
17. #13 OR #14 OR #15 OR #16
18. ((#4 AND #9 AND #12 AND #17) AND 2011:2021[pdat])

**EMBASE (Elsevier)**

1. (‘economic evaluation’ OR ‘cost of illness’):ab,ti
2. (cost-effectiv* OR cost utility OR cost benefit OR cost minimization OR cost*) NEAR/2 (analysis):ab,ti (‘cost-effectiv* analysis’ OR ‘cost utility analysis’ OR ‘cost benefit analysis’ OR ‘cost minimization analysis’ OR ‘costs and cost analysis’):ab,ti
3. ‘economic evaluation’/exp
4. (‘healthcare cost*’ OR ‘health care cost*’ OR ‘economic benfit*’ OR spending OR expenditure OR ‘cost minimiz*’ OR economics):ab,ti
5. ‘health care cost’/de
6. ‘economics’/de
7. #1 OR #2 OR #3 OR #4 OR #5 OR #6
8. (remote NEAR/2 (‘patient monitoring’ OR monitoring OR care OR consultation*)):ab,ti
9. (telemedicine OR telecare OR telehealth OR telenursing OR telemonitoring OR ‘tele monitoring’ OR telemetric OR telehomecare OR ‘tele homecare’ OR telemanagement OR ‘tele management’ OR telerehabilitation OR ‘tele rehabilitation’ OR telesurveillance OR ‘tele surveillance’):ab,ti
10. ‘Telehealth’/exp
11. ‘Telemedicine’/exp
12. ‘Telecare’/exp
13. ‘Telemonitoring’/exp
14. (ehealth OR e-health OR mhealth OR m-health):ab,ti
15. #8 OR #9 OR #10 OR #11 OR #12 OR #13 OR #14
16. (‘home care service*’ OR ‘homecare service*’ OR homecare OR ‘home care’ OR home OR telehomecare OR ‘tele homecare’ OR ‘home telecare’):ab,ti
17. ‘home care’/exp
18. #16 OR #17
19. ((heart OR ‘congestive heart’ OR cardiac) NEAR/2 failure):ab,ti
20. ‘heart failure’/exp
21. (hypertension OR ‘high blood pressure’):ab,ti
22. ‘hypertension’/exp
23. ((heart OR cardiac) NEAR/2 diseas*):ab,ti
24. ‘heart disease’/de
25. (‘heart valve diseas*’ OR ‘heart valve disorder*’ OR ‘valvular heart diseas*’ OR pericarditis OR endocarditis OR myocarditis OR cardiomyopath* OR ‘coronary atherosclerosis’ OR ‘coronary artery atherosclerosis’ OR ‘coronary artery diseas*’ OR ‘pulmonary heart diseas*’ OR ‘cor pulmonale’ OR ‘pulmonary cardiac diseas*’ OR ‘conduction disorder*’ OR ‘cardiac conduction system diseas*’ OR ‘heart muscle conduction disturbance’ OR ‘cardiac dysrhythmia’ OR ‘cardiac arrhythmia*’ OR ‘atrial fibrillation’ OR ‘ventricular fibrillation’):ab,ti
26. ‘valvular heart disease’/exp
27. ‘pericarditis’/exp
28. ‘endocarditis’/exp
29. ‘myocarditis’/exp
30. ‘cardiomyopathy’/exp
31. ‘coronary artery atherosclerosis’/exp
32. ‘cor pulmonale’/exp
33. ‘heart muscle conduction disturbance’/exp
34. ‘heart arrhythmia’/exp
35. (hypertension NEAR/2 ‘heart diseas*’):ab,ti
36. #19 OR #20 OR #21 OR #22 OR #23 OR #24 OR #25 OR #26 OR #27 OR #28 OR #29 OR #30 OR #31 OR #32 OR #33 OR #34 OR #35
37. (#7 AND #15 AND #18 AND #36) AND [2011-2021]/py

**CINAHL (EBSCO)**

1. TI(“economic evaluation” OR “cost of illness)
2. AB(“economic evaluation” OR “cost of illness”)
3. TI((cost-effectiv* OR “cost utility” OR “cost benefit” OR “cost minimization” OR cost*) N2 (analysis))
4. AB((cost-effectiv* OR “cost utility” OR “cost benefit” OR “cost minimization” OR cost*) N2 (analysis))
5. MH(“Costs and Cost Analysis+”)
6. TI(“healthcare cost*” OR “health care cost*” OR “economic benefit*” OR spending OR expenditure OR “cost minimiz*” OR economics)
7. AB(“healthcare cost*” OR “health care cost*” OR “economic benefit*” OR spending OR expenditure OR “cost minimiz*” OR economics)
8. MH(“Health Care Costs+”)
9. S1 OR S2 OR S3 OR S4 OR S5 OR S6 OR S7 OR S8
10. TI((remote) N2 (“patient monitoring OR monitoring OR care OR consultation))
11. AB((remote) N2 (“patient monitoring OR monitoring OR care OR consultation))
12. TI(telemedicine OR telecare OR telehealth OR telenursing OR telemonitoring OR “tele monitoring” OR telemetric OR telehomecare OR “tele homecare” OR telemanagement OR “tele management” OR telerehabilitation OR “tele rehabilitation” OR telesurveillance OR “tele surveillance)
13. AB(telemedicine OR telecare OR telehealth OR telenursing OR telemonitoring OR “tele monitoring” OR telemetric OR telehomecare OR “tele homecare” OR telemanagement OR “tele management” OR telerehabilitation OR “tele rehabilitation” OR telesurveillance OR “tele surveillance)
14. MH(“Telemedicine+”)
15. TI(ehealth OR e-Health OR mhealth OR m-Health)
16. AB(ehealth OR e-Health OR mhealth OR m-Health)
17. S10 OR S11 OR S12 OR S13 OR S14 OR S15 OR S16
18. TI(“home care service*” OR “homecare service*” OR homecare OR “home care” OR home OR telehomecare OR “tele homecare” OR “home telecare”)
19. AB(“home care service*” OR “homecare service*” OR homecare OR “home care” OR home OR telehomecare OR “tele homecare” OR “home telecare”)
20. MH(“Home Health Care+”)
21. S18 OR S19 OR S20
22. TI((heart OR “congestive heart” OR cardiac”) N2 (failure))
23. AB((heart OR “congestive heart” OR cardiac”) N2 (failure))
24. MH(“Heart Failure+”)
25. TI(hypertension OR “high blood pressure”)
26. AB(hypertension OR “high blood pressure”)
27. MH(“Hypertension+”)
28. TI((heart OR cardiac) N2 (diseas*))
29. AB((heart OR cardiac) N2 (diseas*))
30. MH(“Heart Diseases”)
31. TI(“heart valve diseas*” OR “heart valve disorder*” OR “valvular heart diseas*” OR pericarditis OR endocarditis OR myocarditis OR cardiomyopath* OR “coronary atherosclerosis” OR “coronary artery atherosclerosis” OR “coronary artery diseas*” OR “pulmonary heart diseas*” OR “cor pulmonale” OR “pulmonary cardiac diseas*” OR “conduction disorder*” OR “cardiac conduction system diseas*” OR “heart muscle conduction disturbance” OR “cardiac dysrhythmia” OR “cardiac arrhythmia*” OR “atrial fibrillation” OR “ventricular fibrillation”)
32. AB(“heart valve diseas*” OR “heart valve disorder*” OR “valvular heart diseas*” OR pericarditis OR endocarditis OR myocarditis OR cardiomyopath* OR “coronary atherosclerosis” OR “coronary artery atherosclerosis” OR “coronary artery diseas*” OR “pulmonary heart diseas*” OR “cor pulmonale” OR “pulmonary cardiac diseas*” OR “conduction disorder*” OR “cardiac conduction system diseas*” OR “heart muscle conduction disturbance” OR “cardiac dysrhythmia” OR “cardiac arrhythmia*” OR “atrial fibrillation” OR “ventricular fibrillation”)
33. MH(“Heart Valve Diseases+”) OR MH(“Pericarditis+”) OR MH(“Endocarditis+”) OR MH(“Coronary Arteriosclerosis+”) OR MH(“Pulmonary Heart Disease+”) OR MH(“Arrhythmia+”)
34. TI((hypertension) N2 (“heart diseas*”))
35. AB((hypertension) N2 (“heart diseas*”))
36. S22 OR S23 OR S24 OR S25 OR S26 OR S27 OR S28 OR S29 OR S30 OR S31 OR S32 OR S33 OR S34 OR S35
37. S9 AND S17 AND S21 AND S36
38. Publicationdate: 20110101-20211231
39. S37 AND S38

**WEB OF SCIENCE (Clarivate)**

1. TS=(“economic evaluation” OR “cost of illness”)
2. TS=((“cost-effectiv*” OR “cost utility” OR “cost benefit” OR “cost minimization” OR “costs and cost”) NEAR/2 (analysis))
3. TS=(“healthcare cost*” OR “health care cost*” OR “economic benfit*” OR spending OR expenditure OR “cost minimiz*” OR economics)
4. #1 OR #2 OR #3
5. TS=((remote) NEAR/2 (“patient monitoring” OR monitoring OR care OR consultation*))
6. TS=(telemedicine OR telecare OR telehealth OR telenursing OR telemonitoring OR “tele monitoring” OR telemetric OR telehomecare OR “tele homecare” OR telemanagement OR “tele management” OR telerehabilitation OR “tele rehabilitation” OR telesurveillance OR “tele surveillance”)
7. TS=(ehealth OR e-health OR mhealth OR m-health)
8. #5 OR #6 OR #7
9. TS=(“home care services” OR “homecare services” OR homecare OR “home care” OR home OR telehomecare OR “tele homecare” OR “home telecare”)
10. TS=((heart OR “congestive heart” OR cardiac) NEAR/2 (failure))
11. TS=(hypertension OR “high blood pressure”)
12. TS=((heart OR cardiac) NEAR/2 (diseas*))
13. TS=(“heart valve diseas*” OR “heart valve disorder*” OR “valvular heart diseas*” OR pericarditis OR endocarditis OR myocarditis OR cardiomyopath* OR “coronary atherosclerosis” OR “coronary artery atherosclerosis” OR “coronary artery diseas*” OR “pulmonary heart diseas*” OR “cor pulmonale” OR “pulmonary cardiac diseas*” OR “conduction disorder*” OR “cardiac conduction system diseas*” OR “heart muscle conduction disturbance” OR “cardiac dysrhythmia*” OR “cardiac arrhythmia*” OR “atrial fibrillation” OR “ventricular fibrillation”)
14. TS=((hypertension) NEAR/2 (“heart diseas*”)
15. #10 OR #11 OR #12 OR #13 OR #14
16. #4 AND #8 AND #9 AND #15
17. #16 AND PY=(2011-2021)

**SCOPUS (Elsevier)**

1. TITLE-ABS-KEY({economic evaluation} OR {cost of illness})
2. TITLE-ABS-KEY((“cost-effectiv?” OR “cost utility” OR “cost benefit” OR “cost minimization” OR “costs and cost”) W/2 (analysis))
3. TITLE-ABS-KEY(“healthcare cost?” OR “health care cost?” OR “economic benfit?” OR spending OR expenditure OR “cost minimiz?” OR economics)
4. #1 OR #2 OR #3
5. TITLE-ABS-KEY((remote) W/2 (“patient monitoring” OR monitoring OR consultation?))
6. TITLE-ABS-KEY({remote care} OR telemedicine OR telecare OR telehealth OR telenursing OR telemonitoring OR {tele monitoring} OR telemetric OR telehomecare OR {tele homecare} OR telemanagement OR {tele management} OR telerehabilitation OR {tele rehabilitation} OR telesurveillance OR {tele surveillance})
7. TITLE-ABS-KEY(ehealth OR e-health OR mhealth OR m-health)
8. #5 OR #6 OR #7
9. TITLE-ABS-KEY({home care services} OR {homecare services} OR homecare OR {home care} OR home OR telehomecare OR {tele homecare} OR {home telecare})
10. TITLE-ABS-KEY((heart OR “congestive heart” OR cardiac) W/2 (failure))
11. TITLE-ABS-KEY(hypertension OR {high blood pressure})
12. TITLE-ABS-KEY((heart OR cardiac) W/2 (diseas?))
13. TITLE-ABS-KEY(“heart valve diseas?” OR “heart valve disorder?” OR “valvular heart diseas?” OR pericarditis OR endocarditis OR myocarditis OR cardiomyopath? OR {coronary atherosclerosis} OR {coronary artery atherosclerosis} OR “coronary artery diseas?” OR “pulmonary heart diseas?” OR {cor pulmonale} OR “pulmonary cardiac diseas?” OR “conduction disorder?” OR “cardiac conduction system diseas?” OR {heart muscle conduction disturbance} OR “cardiac dysrhythmia?” OR “cardiac arrhythmia?” OR {atrial fibrillation} OR {ventricular fibrillation})
14. TITLE-ABS-KEY((hypertension) W/2 (“heart diseas?”)
15. #10 OR #11 OR #12 OR #13 OR #14
16. #4 AND #8 AND #9 AND #15
17. #16 AND PUBYEAR > 2010

**COCHRANE CENTRAL (Wiley)**

1. (“Economic evaluation” OR “Cost of illness”)ti,ab
2. ((cost-effectiv* OR “cost utility” OR “cost benefit” OR “cost minimization” OR “costs and cost”) NEXT/2 (analysis)):ti,ab
3. [mh “costs and cost analysis”]
4. (“Healthcare cost” OR “Health care cost” OR “Economic benefit” OR Spending OR Expenditure OR “Cost minimization” OR Economics):ti,ab
5. #1 OR #2 OR #3 OR #4
6. ((remote) NEXT/2 (“patient monitoring” OR monitoring OR care OR consultation*)):ti,ab
7. (Telemedicine OR Telecare OR Telehealth OR Telenursing OR Telemonitoring OR “Tele monitoring” OR Telemetric OR Telehomecare OR “Tele homecare” OR Telemanagement OR “Tele management” OR Telerehabilitation OR “tele rehabilitation” OR telesurveillance OR “tele surveillance”):ti,ab
8. [mh “telemedicine”]
9. (Ehealth OR e-Health OR mhealth OR m-Health):ti,ab
10. #6 OR #7 OR #8 OR #9
11. (“home care services” OR “homecare services” OR homecare OR “home care” OR home OR telehomecare OR “tele homecare” OR “home telehealth”):ti,ab
12. [mh “home care services”]
13. #11 OR #12
14. ((heart OR “congestive heart” OR cardiac) NEXT/2 (failure)):ti,ab
15. [mh “heart failure”]
16. (hypertension OR “high blood pressure”):ti,ab
17. [mh “hypertension”]
18. ((heart OR cardiac) NEXT/2 (diseas*)):ti,ab
19. MeSH descriptor: [Heart Diseases] this term only
20. (“heart valve disease” OR “heart valve disorder” OR “valvular heart disease” OR pericarditis OR endocarditis OR myocarditis OR cardiomyopathy* OR “coronary atherosclerosis” OR “coronary artery atherosclerosis” OR “coronary artery disease” OR “pulmonary heart disease” OR “cor pulmonale” OR “pulmonary cardiac disease” OR “conduction disorder” OR “cardiac conduction system disease” OR “heart muscle conduction disturbance” OR “cardiac dysrhythmia” OR “cardiac arrhythmia” OR “atrial fibrillation” OR “ventricular fibrillation”):ti,ab
21. [mh “heart valve diseases”]
22. [mh “pericarditis”]
23. [mh “endocarditis”]
24. [mh “myocarditis”]
25. [mh “cardiomyopathies”]
26. [mh “coronary artery disease”]
27. [mh “pulmonary heart disease”]
28. [mh “cardiac conduction system disease”]
29. [mh “arrhythmias, cardiac”]
30. (hypertension) NEAR/2 (heart diseas*):ti,ab
31. #14 OR #15 OR #16 OR #17 OR #18 or #19 OR #20 OR #21 OR #22 OR #23 OR #24 OR #25 OR #26 OR #27 OR #28 OR #29 OR #30
32. #5 AND #10 AND #13 AND #31
33. Range 2011-2021

**CLINICALTRAILS.GOV**

((“remote patient monitoring” OR telemedicine OR telehealth) AND (“home care services” OR “homecare services” OR homecare OR “home care” OR home OR telehomecare OR “tele homecare” OR “home telecare” OR “home telehealth” )) | (“heart failure” OR hypertension OR “heart disease” OR “heart valve diseases” OR pericarditis OR endocarditis OR myocarditis OR cardiomyopathy OR “conduction disorder” OR “cardiac arrhythmia” OR “atrial fibrillation” OR “ventricular fibrillation”) **|** Start date from 01/01/2011 to 08/19/2021

**NHS EED (York)**

Results for: (((remote patient monitoring OR remote monitoring OR remote care OR remote consultation OR telemedicine OR telecare OR telehealth OR telenursing OR telemonitoring OR tele monitoring OR telemetric OR telehomecare OR tele homecare OR telemanagement OR tele management OR telerehabilitation OR tele rehabilitation OR telesurveillance OR tele surveillance OR ehealth OR e-Health OR mhealth OR m-Health) ) AND ((heart failure OR cardiac failure OR congestive heart failure OR high blood pressure OR hypertension OR heart disease OR cardiac disease OR heart valve diseases OR heart valve disorder OR pericarditis OR endocarditis OR myocarditis OR cardiomyopathy OR cardiomyopathies OR coronary artery disease OR pulmonary heart disease OR cardiac conduction system disease OR conduction disorder OR cardiac dysrhythmia OR cardiac arrhythmia OR atrial fibrillation OR ventricular fibrillation))) and ((Economic evaluation:ZDT and Bibliographic:ZPS) OR (Economic evaluation:ZDT and Abstract:ZPS)) FROM 2011 TO 2021

**COST EFFECTIVENESS ANALYSIS-REGISTRY**

**Note: each set of terms searched individually and all results were downloaded**

1. Remote patient monitoring OR remote monitoring OR remote care OR remote consultation
2. Telemedicine OR telecare OR telehealth OR telenursing OR telemonitoring
3. telemanagement OR telerehabilitation OR telesurveillance OR telemetric OR telehomecare
4. tele monitoring OR tele homecare OR tele management OR tele rehabilitation OR tele surveillance

ehealth OR e-Health OR mhealth OR m-Health

**Supplementary Table 2.** Methodological quality assessment (N = 14).

|  | **Q1** | **Q2** | **Q3** | **Q4** | **Q5** | **Q6** | **Q7** | **Q8** | **Q9** | **Q10** | **Q11** | **Quality of Study** |
| --- | --- | --- | --- | --- | --- | --- | --- | --- | --- | --- | --- | --- |
| **Partial Economic Evaluation** | | | | | | | | | | | | |
| Blum et al.(23) | N | Y | Y | Y | Y | Y | N | N | N | Y | Y | 7/11 |
| Maciejewski et al.(24) | Y | Y | Y | Y | Y | Y | U | N | N | Y | Y | 8/11 |
| Nouryan et al.(25) | N | Y | U | Y | Y | Y | N | N | N | Y | Y | 6/11 |
| Pekmezaris et al.,(22) RCT | N | Y | Y | Y | Y | Y | N | N | N | Y | Y | 7/11 |
| Pekmezaris et al.,(22) QE | N | Y | Y | Y | Y | Y | N | N | N | Y | Y | 7/11 |
| Riley et al.(26) | N | N | U | Y | Y | Y | N | N | N | N | U | 3/11 |
| Wang et al.(27) | Y | Y | Y | Y | Y | Y | N | N | Y | Y | Y | 9/11 |
| White-Williams et al.(35) | N | Y | Y | Y | Y | Y | N | N | N | Y | Y | 7/11 |
| **Full Economic Evaluation** | | | | | | | | | | | | |
| Dehmer et al.(29) | Y | Y | Y | Y | Y | Y | N | Y | Y | Y | Y | 10/11 |
| Fishman et al.(30) | Y | Y | Y | Y | Y | Y | Y | Y | Y | Y | Y | 11/11 |
| Margolis et al.(34) | N | Y | U | Y | Y | Y | Y | Y | Y | Y | Y | 9/11 |
| Martinson et al.(32) | Y | Y | Y | Y | Y | Y | Y | Y | Y | Y | Y | 11/11 |
| Schmier et al.(33) | N | Y | U | Y | Y | Y | Y | Y | Y | Y | Y | 9/11 |
| Willams et al.(31) | Y | Y | Y | Y | Y | Y | N | Y | N | Y | Y | 9/11 |
| **Total %** | 43 | 93 | 71 | 100 | 100 | 100 | 29 | 43 | 43 | 93 | 93 |  |

*Y = Yes; N = No; U = Unclear

†RCT: randomized clinical trial; QE: Quasi-experimental study;

‡JBI critical appraisal checklist for economic evaluations: Q1 = Is there a well-defined question? Q2 = Is there comprehensive description of alternatives? Q3 = Are all important and relevant costs and outcomes for each alternative identified? Q4 = Has clinical effectiveness been established? Q5 = Are costs and outcomes measured accurately? Q6 = Are costs and outcomes valued credibly? Q7 = Are costs and outcomes adjusted for differential timing? Q8 = Is there an incremental analysis of costs and consequences? Q9 = Were sensitivity analyses conducted to investigate uncertainty in estimates of cost or consequences? Q10 = Do study results include all issues of concern to users? Q11 = Are the results generalizable to the setting of interest in the review?

**Supplementary Table 3.** JBI Dominance Ranking Matrix of all included full economic evaluations for RPM with medication management (n = 3).

| Cost | Health benefit | Implication for decision makers | No. of studies |
| --- | --- | --- | --- |
| + | - | Reject intervention |  |
| 0 | - | Reject intervention |  |
| + | 0 | Reject intervention |  |
| - | - | Unclear: judgment required on whether intervention is preferable considering incremental cost effectiveness measures and priorities/willingness to pay |  |
| 0 | 0 | Unclear: judgment required on whether intervention is preferable considering incremental cost effectiveness measures and priorities/willingness to pay |  |
| + | + | Unclear: judgment required on whether intervention is preferable considering incremental cost effectiveness measures and priorities/willingness to pay | 2 (Dehmer et al.,(29) Fishman et al.(30)) |
| - | 0 | Favour intervention |  |
| 0 | + | Favour intervention |  |
| - | + | Favour intervention | 1 (Margolis et al.(34)) |

**Supplementary Table 4.** JBI Dominance Ranking Matrix of all included full economic evaluations in the long run (n = 3).

| Cost | Health benefit | Implication for decision makers | No. of studies |
| --- | --- | --- | --- |
| + | - | Reject intervention |  |
| 0 | - | Reject intervention |  |
| + | 0 | Reject intervention |  |
| - | - | Unclear: judgment required on whether intervention is preferable considering incremental cost effectiveness measures and priorities/willingness to pay |  |
| 0 | 0 | Unclear: judgment required on whether intervention is preferable considering incremental cost effectiveness measures and priorities/willingness to pay |  |
| + | + | Unclear: judgment required on whether intervention is preferable considering incremental cost effectiveness measures and priorities/willingness to pay | 2 (Martinson et al.(32), Schmier et al.(33)) |
| - | 0 | Favour intervention |  |
| 0 | + | Favour intervention |  |
| - | + | Favour intervention | 1 (Margolis et al.(34)) |
